# Linezolid‐Associated Lactic Acidosis Triggering a Sepsis Alert: A Case Report

**DOI:** 10.1002/ccr3.70926

**Published:** 2025-09-22

**Authors:** Yusuf Kagzi, Moni Roy, Tulika Chatterjee

**Affiliations:** ^1^ Department of Internal Medicine University of Illinois College of Medicine Peoria Illinois USA

## Abstract

Elevated lactate alone may trigger false sepsis alerts. In this case, linezolid‐induced lactic acidosis mimicked sepsis, triggering automated alerts. Clinicians should consider medication‐related causes of lactate elevation and apply clinical context in interpreting automated alerts to avoid unnecessary interventions, alert fatigue, and improve diagnostic accuracy in sepsis management.

## 1 Background

Sepsis accounts for around 2 million inpatient stays and nearly 9% of total hospital costs in the United States [1]. In recent years, there has been a 20.1% increase in sepsis‐related inpatient admissions and a 67% increase in aggregate hospital costs. With inpatient sepsis mortality approaching 16.9%, many institutions have incorporated automated best practice advisory (BPA) alerts into their electronic health record (EHR) systems to ensure rapid sepsis management [1]. Serum lactate > 4 mmol/L in sepsis is associated with a 27% mortality rate [2]. A clinical decision support system (CDS) synthesizes data, including lab tests, and prompts a BPA alert if specific criteria are met. However, commission error (overdiagnosis or false positive) and omission error (missed detection or false negative) are standard due to their low positive predictive value. Recent advances using circulating microRNAs (miRNAs) as noninvasive biomarkers may enhance the accuracy of automated sepsis alerts, achieving a specificity of up to 97% [3]. We present a case of isolated lactic acidosis due to linezolid. Linezolid‐induced lactic acidosis is a known side effect of the medication and has been published in the literature. This case highlights a drawback in using fixed‐threshold lactate‐based sepsis alert algorithms, which rely on serum lactate > 2.0 mmol/L without accounting for medication‐induced causes, and the need to integrate additional clinical parameters into alert criteria.

## 2 Case Presentation

A 70‐year‐old obese female with a history of uncontrolled diabetes, chronic obstructive pulmonary disease, and schizophrenia presented with generalized abdominal pain. Pain was intermittent, left‐sided, and associated with non‐bloody emesis, without reported diarrhea, constipation, fever, or chills. The patient had undergone a left great toe amputation for toe osteomyelitis a month before this presentation and was on Linezolid 600 twice daily. Other medications included metformin, insulin, dapagliflozin, atorvastatin, amlodipine, bupropion, risperidone, salmeterol, tiotropium, and fluticasone. The recent amputation site was well healed, with no erythema, drainage, or warmth. On admission, the temperature was 36.4°C, blood pressure 140/84 mmHg, heart rate 83/min, respiratory rate 17/min with O_2_ saturation > 94% on room air. Sepsis BPA alert was fired in the setting of suspected infection and admission lactate of 4.9 mmol/L.

## 3 Investigation, Differential Diagnosis, and Treatment

**FIGURE 1:**
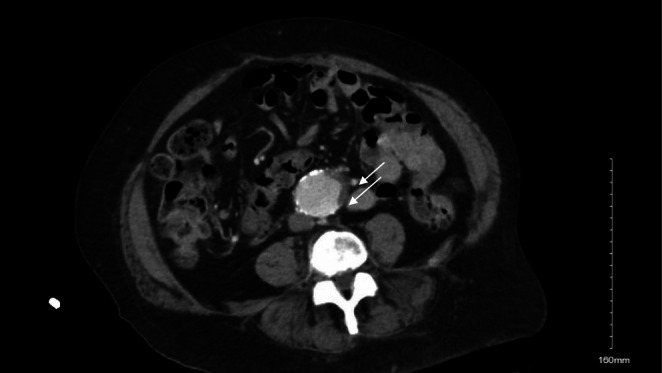
Contrast‐enhanced CT of the abdomen and pelvis demonstrating a stable 3.9 cm infrarenal abdominal aortic aneurysm (white arrow) with no acute intra‐abdominal abnormality, evidence of bowel obstruction, or organized fluid collection.

Initial laboratory evaluation is summarized in Table 1. The patient presented with a serum lactate of 4.9 mmol/L (normal < 2 mmol/L), suggesting lactic acidosis. Renal function was mildly impaired, with a serum creatinine of 1.4 mg/dL and an estimated GFR of 47 mL/min/1.73 m^2^, compared to the patient's baseline creatinine of 1.2–1.3 mg/dL and GFR of 48 mL/min/1.73 m^2^. Metabolic panel showed a serum bicarbonate (CO_2_) of 21 mmol/L, an anion gap of 8 mmol/L, and electrolytes were within the reference range. Stool occult blood and urine analysis were normal. Computed tomographic (CT) abdominal imaging revealed no signs of bowel ischemia, obstruction, or any acute abnormality except a stable 3.9 cm infrarenal aortic aneurysm (Figure 1). Duplex ultrasound for mesenteric arteries and chest X‐ray were unremarkable.

**TABLE 1:** Admission laboratory results.

Parameter	Value	Reference range
White blood cells	5.27 × 10^9^/L	4.5–11.0 × 10^9^/L
Hemoglobin	11.3 g/dL	12–16 g/dL
Platelets	254 × 10^9^/L	150–400 × 10^9^/L
Creatinine	1.4 mg/dL	0.6–1.1 mg/dL
GFR	47 mL/min/1.73 m^2^	> 60 mL/min/1.73 m^2^
Bicarbonate (CO_2_)	21 mmol/L	22–29 mmol/L
Anion gap	8 mmol/L	8–16 mmol/L
C‐reactive protein	1.4 mg/dL	< 1.0 mg/dL
Total bilirubin	0.4 mg/dL	< 1.2 mg/dL
Lipase	24 U/L	< 160 U/L
Random glucose	142 mg/dL	< 140 mg/dL

Sepsis, constipation, ischemic bowel, inadequate flow (hypovolemia, cardiogenic shock, and tissue hypoperfusion), and metformin‐induced lactic acidosis are all potential causes of persistent elevated lactic acid with abdominal pain. Although uncontrolled diabetes can lead to lactic acidosis via dehydration, hypovolemia, or ketoacidosis‐mediated tissue hypoperfusion, our patient maintained near‐normal glycemic control without evidence of diabetic ketoacidosis. Likewise, while severe bowel obstruction or ischemia can elevate lactate through regional hypoperfusion, isolated constipation in the absence of radiographic signs of obstruction, ischemia, or stercoral colitis rarely causes systemic lactic acidosis. These findings and persistent lactate elevations despite correction of volume status and glycemia argue against diabetes or simple constipation as primary drivers.

Given the concern for type B lactic acidosis, metformin was discontinued upon admission, and a bowel regimen was initiated to address possible constipation. Serum lactate levels were closely monitored throughout the hospital stay (Table 2). Although serum lactate modestly improved after metformin was held and intravenous fluids were administered (from 4.9 mmol/L on admission to 2.2 mmol/L at 12 h), levels remained elevated (3.6 mmol/L at 16 h and 2.9 mmol/L at 34 h) while the patient continued on linezolid. On hospital day 3, following suspicion for linezolid‐induced type B lactic acidosis, linezolid was discontinued. Thereafter, lactate declined to 2.1 mmol/L by 30 h post‐discontinuation and, at outpatient follow‐up 7 days later, normalized to 1.8 mmol/L. This temporal association supports linezolid as the likely driver of persistent lactic acidosis in this case.

**TABLE 2:** Serum lactate levels during the course of hospital stay.

Time	On admission	4 h	12 h	16 h	20 h	24 h	30 h	34 h	Day 10
Serum lactate (mmol/L)	4.9	3.1	2.2	3.6	3.1	2.5	2.1	2.9	1.8

## 4 Conclusion and Results (Outcome and Follow‐Up)

Based on prior bone culture for right foot osteomyelitis showing growth of vancomycin‐resistant enterococci (VRE) and methicillin‐resistant *Staphylococcus aureus* (MRSA) and lack of intravenous access due to difficulty in obtaining access and the patient's preference to continue oral antibiotics, linezolid was continued for three more days to complete treatment. She was discharged in stable condition after three additional days of linezolid therapy. At outpatient follow‐up, her lactate level had normalized (1.8 mmol/L).

## 5 Discussion

Finding an acceptable trade‐off between reliability and sensitivity in a sepsis BPA alert can be difficult. BPA is a real‐time clinical decision support that continuously monitors patient data, including vital signs, laboratory values, and other clinical indicators, and triggers an alert when predefined sepsis criteria are met.

As per the Society of Critical Care Medicine, automated sepsis screening tools in EHR use systemic inflammatory response syndrome (SIRS) criteria, vital signs, signs of infection, quick Sequential Organ Failure Score (qSOFA), National Early Warning Score (NEWS), or Modified Early Warning Score (MEWS), with the majority having poor predictive values [4, 5]. These tools use advanced algorithms in real time that are highly variable and complex, with most systems assessing some standard variables [6] (Table 3). A new early recognition tool at our level 1 teaching hospital, the sepsis alert system, is powered by a predictive modeling tool integrated into the EHR. This system uses two models: a Pre‐Lab model that evaluates patient data (e.g., vital signs, medical history, and triage details) before laboratory results are available, and a Post‐Lab model that incorporates lab values such as white blood cell count, lactate, and creatinine once they are reported. The models generate a real‐time sepsis risk score that updates every 15 min.

**TABLE 3:** Major criteria incorporated within the automated sepsis alert systems (Hwang et al., 2020) [7].

**Major criteria in the threshold of the automated sepsis alert systems**
SIRS criteria: Temperature > 38.3 or < 36.0°C, HR > 90/min, RR > 20/min, WBC > 12,000 or < 4000 per microliter of blood or > 10% bands on complete blood count (CBC) Acute organ dysfunction (within the last 6 h.): SBP < 90 or a decrease of > 40 mmHg or non‐fluid responsive hypotension MAP < 65 mmHg Lactate ≥ 2 (mmol/L) Creatinine > 2 mg/dL Total bilirubin > 2 mg/dL Platelet count < 100,000 per microliter of blood INR > 1.5 or aPTT > 60 s Acute respiratory failure, or Elevated troponin levels Glasgow coma score or new signs of altered mental status Suspected or known infection source Demographics with patient history and already ordered lab results Sepsis risk factors such as diabetes, immunosuppression, chronic renal, liver, or cardiac illness

Abbreviations: aPTT, activated partial thromboplastin time; INR, international normalized ratio; mg/dL, milligrams per deciliter; mmHg, millimeters of mercury; mmol/L, millimoles per liter.

A BPA sepsis alert is triggered if the patient's score crosses a predetermined threshold. Once activated, the BPA delivers a pop‐up notification to clinicians within the EHR, prompting the clinical team to reassess the patient and suggesting actions such as ordering diagnostic tests, initiating sepsis protocols, or starting empirical antibiotics. Although these tools aim to expedite early identification and management of sepsis, they have a sensitivity of 31% with a precision of 59% [6, 7]. It may also contribute to alert fatigue when triggered by noninfectious causes such as isolated lactate elevation [8]. In our case, a sepsis alert was fired due to high lactate and suspected infection, without any other evident clinical signs of sepsis (Table 4). Our patient did not meet SIRS criteria, with no fever, tachycardia, leukocytosis, or hypotension, and imaging showing no clear source of infection. Despite stopping metformin and administering fluids, lactate levels remained elevated during ongoing linezolid therapy. A sustained drop in lactate occurred only after linezolid was discontinued, with normalization noted at outpatient follow‐up. This temporal association, combined with the known mitochondrial toxicity of linezolid and the absence of alternative causes, strongly supported linezolid‐induced type B lactic acidosis as highlighted in our case.

**TABLE 4:** Patient findings vs. sepsis alert criteria.

Sepsis alert criterion	Threshold	Patient's finding	Meets criterion?
Temperature	> 38.3°C or < 36.0°C	36.4°C	No
Heart rate (HR)	> 90/min	84 bpm	No
Respiratory rate (RR)	> 20/min	17 breaths/min	No
White blood cell count (WBC)	> 12,000 or < 4000 or > 10% bands	5270, bands < 10%	No
Lactate	≥ 2 mmol/L	4.9 mmol/L	Yes
Systolic (BP mmHg)/MAP	SBP < 90/MAP < 65	SBP 140/MAP > 65	No
Creatinine	> 2.0 mg/dL	1.4 mg/dL	No
Total bilirubin	> 2.0 mg/dL	0.4 mg/dL	No
Platelets	< 100,000/μL	> 100,000/μL	No
INR/aPTT	INR > 1.5 or aPTT > 60 s	Normal	No
Altered mental status/GCS	New change in consciousness, awareness, cognition, or behavior	Absent	No
Infection source	—	Absent	No
Risk factors	Diabetes, immunosuppression, chronic renal, liver, or cardiac illness	Diabetes	Yes

Serum lactate > 2.0 mmol/L is considered a sign of organ dysfunction and fires sepsis alert, whereas levels > 4.0 mmol/L indicate septic shock [8]. An institutional study suggests that early lactate (normal: 1.6–2.5 mmol/L) testing in the emergency department may increase the number of patients identified. However, this may not necessarily mean more patients benefit from early antibiotics and aggressive resuscitation [9]. Due to low sensitivity, serum lactate is not recommended as an adjunctive test to modify the pretest probability of sepsis [4].

Although serum lactate is an important indicator of tissue hypoxia, it does not directly evaluate tissue perfusion. Type A lactic acidosis occurs due to decreased perfusion secondary to hypovolemia and sepsis. In contrast, type B occurs due to impaired cellular metabolism, such as drug use like metformin, linezolid, propofol, beta‐agonist, and nucleoside reverse transcriptase inhibitors. The cause of lactic acidosis in sepsis is mitochondrial dysfunction in the lungs. On the other hand, metformin increases lactate through intestinal transformation and hepatic mitochondrial metabolism suppression. Linezolid, an oxazolidinone antibiotic, inhibits respiratory chain activity containing mitochondrial DNA. Incidence for lactic acidosis among individual drugs is < 5%; however, concurrent use of LZD and metformin increases the risk of lactic acidosis [3]. Risk is further increased with renal insufficiency, duration, and dose of drug [10].

Alerts are intended to be a decision tool to reevaluate a patient's current clinical status rather than diagnosis [11]. Moreover, overidentification and alert fatigue would diminish the utility of such warnings. Lactate alone is neither sensitive nor specific enough to rule in or rule out the diagnosis on its own. Other recommended parameters, including capillary refill time, mean arterial pressure (MAP), signs of infections, and procalcitonin, can increase sensitivity and reduce commission error [4]. Although persistently elevated lactate levels predict a high mortality risk, cases of isolated raised lactate should be interpreted in a correct clinical context, and other causes such as medications, diabetes, trauma, toxins, and cardiac or liver disease should be considered [12]. In patients at elevated risk for lactic acidosis, alternative agents to linezolid should be considered for VRE/MRSA coverage. Daptomycin, a cyclic lipopeptide, offers once‐daily bactericidal activity against VRE/MRSA (excluding pneumonia), while tigecycline covers both but has limited use in bloodstream infections [13, 14]. Tedizolid, a newer oxazolidinone, may carry a lower toxicity profile than linezolid, though data on lactic acidosis are scarce. However, because these agents are available only as intravenous formulations, and our patient had limited IV access and preferred to continue oral therapy, none were feasible options in this case [15].

While early sepsis resuscitation focuses on prompt infection identification to initiate appropriate antibiotics with aggressive fluid hydration, drug‐induced lactic acidosis treatment should focus on identifying the culprit drug and possible discontinuation of such a drug after risk vs. benefit discussion with the patient. When treating lactic acidosis, the selection of crystalloids should be based on the etiology of lactic acidosis, as crystalloids such as Ringer's lactate may worsen type‐B lactic acidosis and unnecessary antibiotic use [16, 17]. Overall, with an in‐depth clinical assessment, false automated alerts can be considerably decreased, lowering costs, reducing alert fatigue, and enhancing overall system efficacy.

This report describes a single patient's experience at one institution, limiting our observations. Additionally, institutional differences in alert thresholds, laboratory turnaround times, and clinical workflows may affect the applicability of our findings to other settings. Further studies are needed to validate the impact of incorporating medication history into sepsis alert algorithms and determine optimal alert thresholds that balance sensitivity and specificity.

## Author Contributions

**Yusuf Kagzi:** conceptualization, formal analysis, investigation, writing – original draft. **Moni Roy:** conceptualization, formal analysis, investigation, supervision, validation, writing – review and editing. **Tulika Chatterjee:** conceptualization, validation, writing – review and editing.

## Consent

Written consent was collected from the next of kin to publish this report and is available upon request by the journal's editor.

## Conflicts of Interest

The authors declare no conflicts of interest.

## Data Availability

Data supporting the conclusions of this report is contained, and nonrelevant patient data are protected under patient privacy regulations and policies.
